# Social and Poor-Law Problems

**Published:** 1906-07-14

**Authors:** 


### SOCIAL AND POOR LAW PROBLEMS.

### THE VAGRANTS' TASK.

The amount of work that ought to be demanded from a tramp in payment of his bed and breakfast in a casual ward has been occupying the attention of several Boards of Guardians lately. At Cuckfield the assent of the Local Government Board has been obtained to a proposal that the male casual should be asked to pound only 1 cwt. of stone, while at Halifax the task is 7 cwt., and at Worcester the maximum task for able-bodied men in the workhouse is 13 cwt., but the master is allowed to use his discretion as to imposing so large a task, so that those who are not in a good state of health should not be overburdened. The enormous disproportion of the task set in these different places makes one doubt the usefulness of local authority. Are the casuals at Cuckfield such a delicate race that they cannot reasonably be asked to do as much as those of Halifax—not to speak of Worcester? It would be interesting to compare the records of the casual wards of all three places a year hence, and see to what extent the light task has attracted vagrants and the heavy one kept them away. As to what constitutes a reasonable task, it is not easy to say. Perhaps the only satisfactory way to decide the point is that adopted by the chairman and clerk of the Halifax Guardians, who had themselves shut into two cells in the casual ward, measuring each 6 feet by 8 feet, and locked in these, set upon the task usually given to vagrants. At the end of three hours, when the gentlemen were released, the chairman had an hour's work to do, and the clerk about twice as much. To men untrained to manual labour, therefore, the breaking of 7 cwt. of granite means between four and five hours' work. But it is doubtful if it would take an experienced tramp so long. At any rate the experiment did not make the investigators think that it was necessary to reduce the task at present set. They made some discoveries, however, that will probably lead to changes. One was that the cells were rather small for convenience of work, and that they needed greater facilities for the escape of dust. This is rather an important point, for no one desires that task-work of this sort should be unhealthy, as it may be if the dust raised by the stone-breaking does not escape freely. It is unlikely that the amount set is excessive, as the quantity to be broken by a criminal in prison, where the physical effects of such labour can be more closely watched than in a casual ward, is 8 cwt. As for the 13 cwt. which the Worcester pauper is to tackle, it is avowedly meant to drive out those able-bodied loafers who will spend all their lives in a workhouse unless the conditions there are made harder than those of the world outside. The principle of making the lot of the pauper less attractive than that of the free worker is undoubtedly sound, and it is to be hoped that the discretion allowed to the master in fixing the task will prevent hardship to those who are old and weakly.

